# Personality predicts collective behavior in greylag geese: Influencers are bold and followers are exploratory

**DOI:** 10.1016/j.isci.2025.113170

**Published:** 2025-07-22

**Authors:** Sonia Kleindorfer, Andrew C. Katsis, Didone Frigerio, Jonas Lesigang, Dina Mostafa, Lauren K. Common

**Affiliations:** 1 Konrad Lorenz Research Center for Behavior and Cognition, Core Facility of the University of Vienna, Grünau im Almtal 4645, Austria; 2 Department of Behavioral and Cognitive Biology, University of Vienna, Vienna 1030, Austria; 3 College of Science and Engineering, Flinders University, Adelaide, SA 5042, Australia; 4 Department of Biology, University of Copenhagen, 2100 Copenhagen, Denmark

**Keywords:** Wildlife behavior, Zoology, Ornithology, Social sciences, Psychology

## Abstract

Simple interaction rules describe the coordination of individual behavior into collective behavior. However, we lack long-term tests of individually tagged individuals in the wild to understand fitness payoffs of different social roles during collective movement. Here, we interrogate leader-follower roles in greylag geese (*Anser anser*) in relation to personality traits (boldness, aggressiveness, and exploration). We calculated an influencer score based on the number of followers for sub-group movement events across four years. Influencer score was weakly but significantly repeatable over time, and all three personality traits were repeatable. Greylag geese with higher influencer scores were bolder, and geese that were first to follow a non-partner influencer goose were more exploratory and more likely to look behind the mirror during a mirror stimulation test. In light of these findings, we discuss potential individual-level benefits of following a bold individual and the presumed group-level benefits of new information spread via followers open to novelty.

## Highlights

• Personality traits predict roles in collective movement of greylag geese
• Exploratory geese follow bold leaders, who attract more followers
• Aggressiveness shows no link to leadership or following roles
• Influencer roles remain consistent across years

## Graphical abstract

**Figure:**
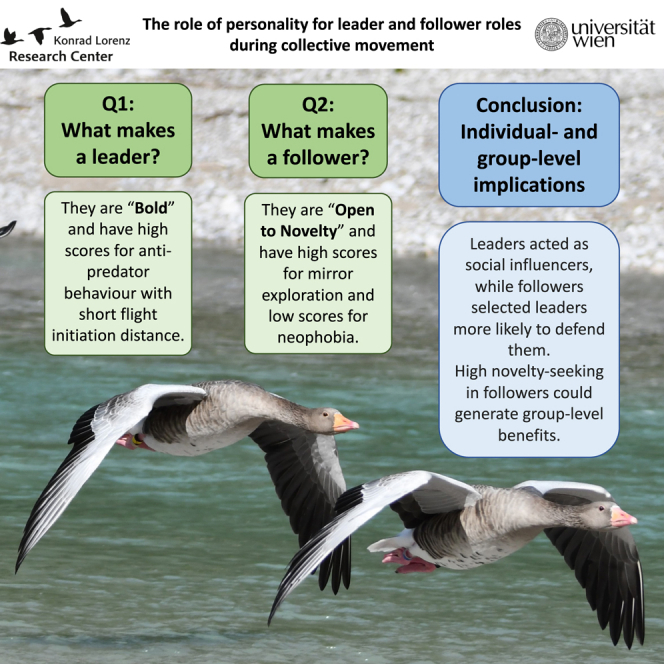

## Introduction

The coordination of individual behavior into collective behavior may follow simple interaction rules, yet is theoretically challenging to explain because individuals experience different costs and benefits of collective group decisions.^1^^,^^2^ In free-living primates such as baboons, the process of collective movement is underpinned by mechanisms for consensus reaching, suggesting that so-called democratic processes can describe some forms of collective behavior.^3^ Despite shared decision-making in wild baboons, there is also evidence for robust individual roles in leader-follower dynamics, whereby some individuals are more likely to be followed than others.^4^ Generally, there has been more focus on the attributes of the (emergent) leader in consideration of collective group decision-making,^5^ with less attention paid to the attributes of followers (but see studies by Webster et al.^6^ and Kummer^7^). Understanding how individual phenotypic variation influences the composition of a group may elucidate some puzzling aspects of collective behavior, such as why some individuals wield more influence than others under some conditions.

Past studies have identified a variety of traits, such as experience, sex, age, and social dominance, that affect individuals’ propensity to exert influence on group-level decisions.^8^^,^^9^^,^^10^ Independently of the interaction rules that can “blindly” produce an emergent leader, individuals may attempt to influence other group members and thereby shape group-level decisions.^1^ More recently, a body of evidence supporting individual personality differences^11^ is being applied to examine leader and follower roles.^12^^,^^13^^,^^14^ The useful perspective provided by personality research in the context of collective decision-making is a better understanding of the costs and benefits to individuals of following or not following others with specific personality traits. This perspective does not focus on how a decision is made, but rather on the adaptive context of why a particular individual may be more likely to be followed or to be an influencer.

The study of individual differences can reveal selection pathways for personality traits associated with collective behavior.^15^ One outcome of collective movement can be access to new information,^16^ for which the fitness payoff may differ between individuals. For example, ordered or disordered group structures can alter the payoff to first-arriving individuals at a new food source.^17^ Likewise, the predation risk of following certain individuals over others would carry different costs when following an inexperienced individual,^18^ an unreliable individual,^19^ or a bold individual who defends the followers against predators.^20^ Natural selection is expected to favor individuals that can discriminate conspecific behavioral traits that confer a fitness benefit to the discerner.^21^^,^^22^

The flock of greylag geese (*Anser anser*) at the Konrad Lorenz Research Center in Grünau im Almtal have been color banded and studied since 1952—and since 1973 at their current site—filling an important gap in studies of collective behavior in the wild: flock members are individually identifiable, and we have “nearly complete” life history records for each goose since the flock’s inception. Building on these life history data and with the addition of new research on individual differences, we can better understand the link between individual differences, social roles, and fitness outcomes.^23^^,^^24^ Greylag geese have individually distinctive faces^25^ and calls^26^ and possess a range of cognitive abilities that support the view that they can discriminate between individual conspecifics.^27^^,^^28^ In this study, we ask if greylag geese have consistent influencer roles (number of followers) and whether certain personality traits are associated with occupying an influencer or follower role. Although greylag geese associate in flocks of 100 individuals or more, individuals moving between foraging sites typically depart in smaller sub-groups. Flight departure is preceded by a predictable succession of behaviors. Up to half an hour before taking flight, individuals start to position themselves in the direction of departure.^29^ While this orienting behavior occurs, individuals begin to produce a recruitment call—a repetitive staccato call produced in long bouts of the same syllable.^30^ This is followed by the departure call, a high-amplitude single-element vocalization that signals that goose’s imminent departure.^30^

To determine which individuals were influencing sub-group departures, we used focal observations of flock sub-groups to record (1) the identity of the individual that first produced departure calls within that sub-group; (2) departure group size, the number of geese (if any) that flew together within 2 min of that individual’s departure calls (including partners and fledglings); and (3) the identity of the goose that was first to follow the departure caller after it took flight. If the focal goose did not depart within 2 min of its own departure calling, then departure group size was recorded as 0; if the focal goose departed alone, then departure group size was recorded as 1. For each individual that first produced departure calls, we used departure group size as its “influencer score”, a metric of group leadership. Next, we used a standardized battery of personality assays to measure boldness (flight initiation distance, FID, in response to a human approach), aggressiveness (mirror stimulation test), and exploration (neophobia during a novel object test). We ask: (1) is there repeatability in an individual’s influencer score across years? And (2) are there effects of life history (sex, age, pairing status, and family size) and personality (boldness, aggressiveness, and exploration) on influencer score and the frequency of follower events?

## Results

### Repeatability of influencer score and personality

Once an individual initiated departure calling within its sub-group, we recorded the number of geese that flew together within the next 2 min, which we recorded as its influencer score. An individual’s influencer score was significantly repeatable, although repeatability was low (adjusted R = 0.09 ± 0.03, 95% confidence intervals (CIs) = 0.03–0.16, *p* < 0.001). In addition, we measured three personality traits on multiple occasions, all of which were significantly repeatable (boldness: adjusted R = 0.12 ± 0.03, 95% CIs = 0.06–0.18, *p* < 0.001; aggressiveness: adjusted R = 0.34 ± 0.12, 95% CIs = 0.09–0.54, *p* = 0.003; neophobia: adjusted R = 0.48 ± 0.07, 95% CIs = 0.33–0.60, *p* < 0.001).

### Life history, personality, and influencer score

**Figure 1:**
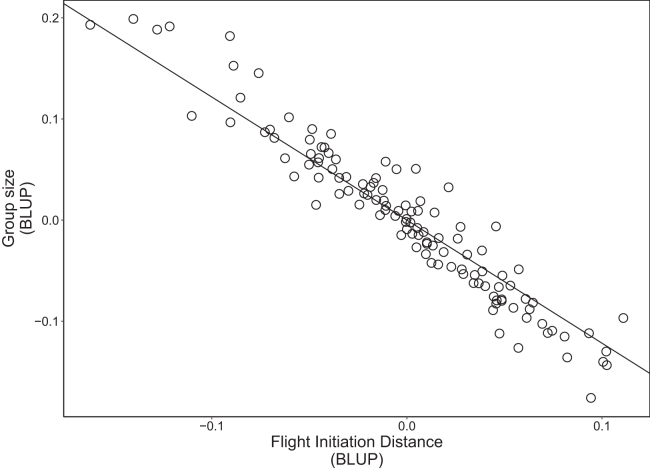
Association between an individual’s boldness and influencer score Best linear unbiased predictors (BLUPs) from a bivariate linear mixed model showing the among-individual correlation between an individual’s boldness (flight initiation distance) and influencer score (departure group size). Bolder individuals (shorter flight initiation distance) recruited a larger group size following their departure calls. See also Table S1.

**Figure 2:**
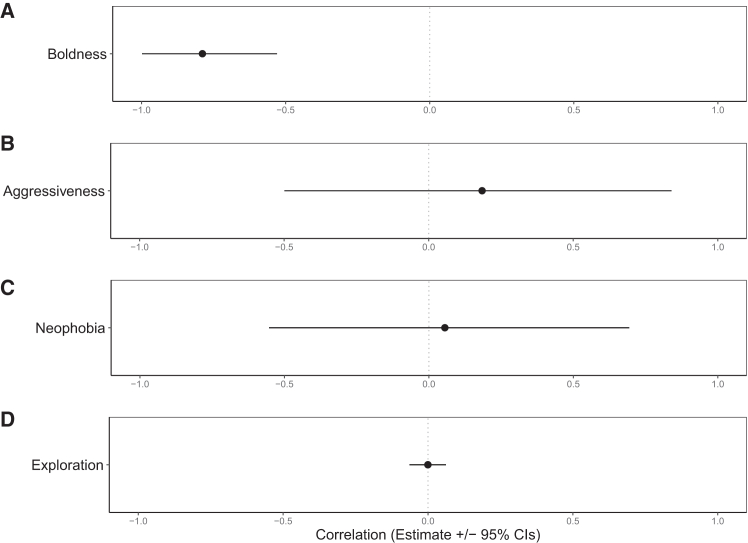
Personality traits and influencer group size in greylag geese Estimated correlations (mean ± 95% CrI) between focal individuals’ departure group size after departure calling and four behavioral traits: boldness (flight initiation distance during human approach assays), aggressiveness (response to a mirror stimulation test), neophobia (response to a novel object test), and mirror inspection (looked behind the mirror during a mirror stimulation test). Credible intervals do not overlap zero for boldness, indicating that bolder geese (i.e., with shorter flight initiation distances) recruited more followers. See also Table S1.

**Figure 3:**
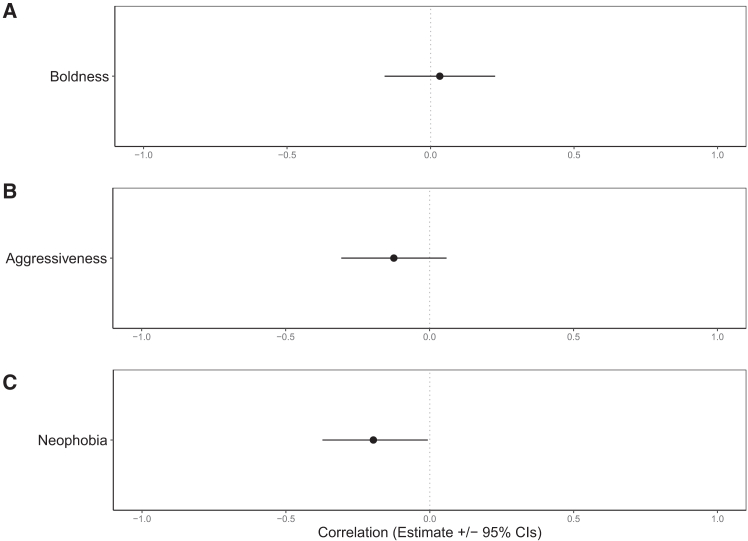
Personality traits associated with first-follower behavior in greylag geese Estimated correlations (mean ± 95% CrI) between an individual’s first-follower count (the number of times it was first to follow a departing goose) and three personality traits: (A) boldness (flight initiation distance during a human approach assay), (B) aggressiveness (response to a mirror stimulation test), and (C) neophobia (response to a novel object test). Credible intervals do not overlap zero for neophobia, indicating that less neophobic geese (i.e., with shorter latency from 2 m to 1 m to approach a novel object) were more likely to be the first to follow a non-partner departing goose. See also Table S2.

We tested which life history and behavioral variables are associated with an individual’s influencer score. The sex of the bird did not predict its influencer score (estimate = −0.03 ± 0.04, *p* = 0.508), nor did its family size (including partner and fledglings: estimate = −0.02 ± 0.02, *p* = 0.497); however, older and paired individuals had more followers compared to younger and unpaired individuals, respectively (hatch year: estimate = −0.05 ± 0.02, *p* = 0.010; pairing status: estimate = −0.19 ± 0.07, *p* = 0.004; Table 1; Figure S1). Year had a significant effect on influencer score (χ^2^ = 20.67, *p* < 0.001; Table 1; Figure S1), but there was no effect of month (estimate = −0.13 ± 0.11, *p* = 0.246, Table 1). Boldness was significantly correlated with influencer score, with bolder birds (i.e., those with shorter FIDs) recruiting more followers upon their departure (correlation estimate = −0.79, 95% credible intervals [CrIs] = −0.99, −0.52; Table S1; Figures 1 and 2). Aggressiveness, neophobia, and mirror inspection (whether a goose looked behind the mirror during the mirror stimulation test) were not significantly correlated with influencer scores (Table S1; Figure 3).

**Table 1:** Linear mixed model results for factors affecting influencer group size after departure calling in greylag geese

	Estimate	SE	df	T	ChiSq	P
Intercept	0.67	0.09	655.44	7.32		
Month	−0.13	0.11	717.18	−1.16	1.35	0.246
Year (2021)^a^	0.10	0.05	685.51	1.97	20.67	**<0.001**
Year (2023)^a^	−0.06	0.05	718.30	−1.26		
Focal Sex^b^	−0.03	0.04	93.87	−0.66	0.43	0.508
Family Size	−0.02	0.02	303.73	−0.68	0.46	0.497
Hatch Year	−0.05	0.02	61.34	−2.58	6.67	**0.010**
Focal pairing status^c^	−0.20	0.07	159.92	−2.91	8.49	**0.004**

Output from a linear mixed model testing the effects of observation month, observation year, focal sex, family size, hatch year, and pairing status on departure group size after departure calling in greylag geese. See also Figures S1, S2, and S3. Departure group size was log-transformed. n = 742 observations for 117 individuals. Pairing status was either paired or unpaired. Random effect individual ID variance 0.01 ± 0.11. Bold values indicate statistical significance (*p* < 0.05).

a 2020 used as reference category.

b Female used as reference category.

c Paired used as reference category.

### Life history, personality, and first-to-follow count

A bird’s sex, age, and pairing status significantly predicted the number of times it was recorded as being the first to follow a departing goose (sex: estimate = −0.73 ± 0.27, *p* = 0.008; hatch year: estimate = −0.49 ± 0.15, *p* = 0.002; pairing status: estimate = −1.09 ± 0.54, *p* = 0.047; Table 2; Figure S2). Specifically, first-followers were more often female, young, and paired, respectively (Figure S2). There was no effect of family size on the likelihood of being first to follow a departing goose (estimate = −0.85 ± 0.48, *p* = 0.075, Table 2).

**Table 2:** Linear model results for factors affecting the number of times a goose was recorded as first follower

	Estimate	SE	T	SumSq	F	P
Intercept	1.64	0.25	6.65			
Focal sex^a^	−0.73	0.27	−2.72	13.65	7.38	**0.008**
Family size	−0.85	0.48	−1.80	5.97	3.23	0.075
Hatch year	−0.49	0.15	−3.16	18.44	9.97	**0.002**
Focal pairing status^b^	−1.09	0.54	−2.01	7.47	4.04	**0.047**

Output from a linear model testing the effects of focal sex, hatch year, and pairing status on the number of times a goose was recorded as being the first to follow a departing goose. See also Figure S2. *n*= 117 individuals between 2020 and 2023. Pairing status was either paired or unpaired. Bold values indicate statistical significance (*p* < 0.05).

a Female used as reference category.

b Paired used as reference category.

**Figure 4:**
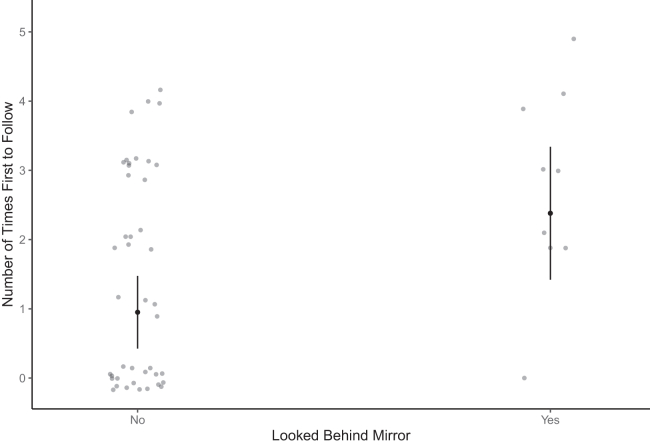
Mirror inspection behavior and first-follower frequency in greylag geese The relationship between mirror inspection behavior (whether or not a goose looked behind the mirror during the mirror stimulation test) and the number of times a goose was recorded as being the first to follow throughout the study period (2020–2023, n = 51 individuals). Error bars represent 95% CIs around the estimated marginal mean. See also Table S3.

There were no significant relationships between an individual’s boldness or aggressiveness and the number of times they were first to follow a departing goose (Table S2). However, there was a significant effect for neophobia (correlation estimate = −0.20; 95% CrIs = −0.37, −0.01; Table S2), with less neophobic birds being first to follow more often (Table S2; Figure 2). Mirror inspection behavior during the mirror stimulation test significantly predicted the number of times a goose was first to follow a departing goose; that is, individuals that looked behind the mirror were more often first to follow than individuals that did not look behind the mirror (estimate = 1.43 ± 0.51, *p* = 0.007, Table S3; Figure 4).

## Discussion

Greylag geese had low but statistically significant repeatability in their influencer score measured across four years in 117 greylag geese. All three personality traits—boldness, aggressiveness, and exploration (neophobia)—were weakly to moderately repeatable among individuals, with our estimates for aggressiveness (0.34) and exploration (0.48) comparable to the mean repeatability of behavioral traits (0.37) in a prior meta-analysis.^31^ Bolder greylag geese had higher influencer scores, meaning that more geese followed them into flight. Geese that were first to follow another goose’s departure were more likely to exhibit mirror inspection behavior (looking behind the mirror) during a mirror stimulation test and were also less neophobic toward a novel object. Neither influencer nor first-follower geese had high scores for aggressiveness.

Influencer scores were weakly but significantly repeatable over time, which suggests that some flock members are consistently recruiting more followers and, hence, are favored as sub-group leaders. However, the relatively low repeatability estimate in our study (R = 0.08) indicates that factors other than individual identity are also at play. Previous work in swans and geese has shown that variation in pre-flight signals can affect departure group size, with individuals that signal for longer before departure recruiting more followers.^32^^,^^33^ In turn, the time dedicated to pre-flight signaling may vary with transient factors, such as partner proximity, level of hunger, or the expected food reward at their next destination.^32^ Nevertheless, our finding that leader and first-follower roles remained significantly repeatable across four years suggests that this finding is robust to potential annual and seasonal effects.

An individual’s influencer score is also seemingly shaped by its behavioral phenotype, given that bolder geese (i.e., those with shorter flight initiation distances) had more flockmates following them into flight. From the perspective of individual-level decision-making by follower geese, following a bold rather than aggressive goose potentially provides fitness benefits via increased defense against predators and lower costs of agonistic competition over food resources at their next destination.^34^ Conversely, departing with an aggressive goose would presumably result in less food acquisition for the follower, as found in other taxa,^35^^,^^36^^,^^37^ with the aggressive individuals tending to out-compete their flockmates. In Arctic barnacle geese (*Branta leucopsis*), subordinate geese were more exploratory, and these more exploratory geese were faster to discover new foraging sources—only to be quickly supplanted by dominant geese.^38^

In this study, geese that were less neophobic toward a novel object were more likely to be first followers in a departure event. Neophobia is often interpreted as a measure of exploration,^11^ with less neophobic (more exploratory) individuals in this study tolerating greater novelty while foraging. This has also been reported in homing pigeons (*Columba livia*), where more exploratory individuals in a novel environment test covered more of their local area during spontaneous exploration flights and took more direct routes home when released far afield, perhaps due to greater familiarity with the landscape.^39^ Hence, exploratory geese in a departing subgroup potentially play a key role in their group’s navigation and landscape use (e.g., promoting the discovery of novel foraging sites) and in the transmission of new information, including feeding innovation (e.g., novel plants) that may contribute to the spread of new feeding traditions in the group.^40^ By contrast, sub-groups composed of aggressive leaders and followers may be more likely to maintain the status quo, with decision-making dominated by the aggressive leader. Resource acquisition and spatial distribution in greylag geese seem to occur across two pathways: resource access mediated by aggressiveness and dominance rank on the one hand, and information acquisition, and perhaps information spread, mediated by boldness and exploration on the other hand.^41^^,^^42^ It remains to be tested how information acquisition and spread affect the likelihood of innovation and the establishment of new traditions.^43^^,^^44^ A recent study of barnacle geese documented the rapid emergence of a new migratory and foraging tradition led by older birds, who were presumably more experienced and inspired more followers.^45^ This highlights the importance of social processes for determining leaders and followers, which may in turn influence a population’s capacity to establish new foraging or nesting areas in the face of environmental change.^45^ In our study, geese that looked behind the mirror during the mirror stimulation test were also more likely to be first followers, although interpreting this behavior is more difficult. Some studies treat mirror inspection as a type of exploration,^46^^,^^47^ which would again indicate an individual’s tendency to search for food in novel locations; however, more work is needed to confirm the behavioral and/or cognitive mechanisms underlying this behavior. In particular, the presence of a conspecific stimulus (i.e., the focal goose’s mirror-image) complicates how we interpret individual responses to this assay, which may incorporate aspects of exploration, aggressiveness, and sociability.

Focusing on the association between personality and social roles such as influencer or follower does not illuminate the mechanisms of group decision-making, only its presumed adaptive benefits. An exploratory (open-minded) follower that chooses to follow a bold leader will likely derive protection benefits, perhaps also via the benefits of a mixed network, as seen in other contexts.^48^ These findings contribute to a growing body of research into possible selection pathways shaping the benefits of individual differences, and hence phenotypic variation and heterogeneity, in groups.^49^^,^^50^^,^^51^ Group decision-making processes that facilitate the transmission of information (e.g., through social learning) and the movement behavior of populations can potentially give rise to different migration cultures.^52^ A better understanding of how individual social roles facilitate the transmission of information, which can promote adaptive behavior and shape migration culture, will increase our knowledge of the adaptability of populations to extreme environmental conditions.

The strong individuality signatures in greylag geese in their physical appearance^25^ and vocalizations,^26^ combined with their capacity for transitive inference,^28^ gaze following,^27^ social imitation,^53^ and stimulus enhancement learning,^40^ underscore particular aspects of this system that promote individuality signaling and/or individuality cues, such as individual personalities. The cognitive capacity to predict the behavior of a group member in a novel or threatening situation would presumably be a factor that co-influences patterns of association, including influencer and follower roles. It is also possible that personality traits interact with social feedback mechanisms in a way that enhances these effects: for example, bolder leaders may attract more or more faithful followers, which in turn might induce stronger leadership behavior.^54^ Hence, the role of personality traits in the creation and maintenance of leader-follower roles need not replace more traditional models of social feedback but may instead act in concert with them.^13^ This interplay has been observed, for example, in barnacle geese, where more exploratory individuals influenced decision-making in a group navigation task but this influence diminished as group size increased.^55^ Such findings underscore the idea that social learning processes are important for the adaptation of populations to environmental changes,^52^ especially under conditions of rapid and extreme climate change.^45^

Previous research has focused heavily on the attributes of leaders, implying that leaders exert influence on followers. The findings presented here point to a stronger role of the cognitive capacity of followers to predict beneficial attributes of individuals they choose to follow. As foreshadowed by Kummer,^7^ increased focus on the attributes of followers, including their openness to and dissemination of new information, perhaps via social imitation and other mechanisms, is a fruitful area of future research. In this way, the group-level benefits of phenotypic heterogeneity come into a new light.

### Limitations of the study

While our study provides insights into leadership and personality traits in greylag geese, several limitations should be acknowledged. First, inferring leadership dynamics in free-ranging groups remains challenging due to the complexity of collective decision-making processes and potential context dependency of leadership roles.^1^^,^^2^ Leadership may not be fixed but can be flexible and shared among individuals depending on environmental and social factors.^4^^,^^5^ Additionally, personality traits such as boldness and exploration influence social interactions and leadership tendencies but interact in complex ways with social feedback and experience.^12^^,^^13^ Methodologically, limitations in tracking fine-scale interactions and disentangling causality between personality and leadership remain.^3^^,^^15^ Finally, while we interpret behavioral patterns within an evolutionary ecology framework, the generalizability of findings to other species or ecological contexts requires caution.^11^^,^^14^

## Resource availability

### Lead contact

Further information and requests for resources should be directed to and will be fulfilled by the lead contact, Sonia Kleindorfer (sonia.kleindorfer@univie.ac.at).

### Materials availability

This study did not generate new reagents.

### Data and code availability

• Data have been deposited in the PHAIDRA: 10.25365/phaidra.510, and are publicly available as of the date of publication.
• All original code is available in this paper’s supplemental information.
• Any additional information required to reanalyze the data reported in this paper is available from the lead contact upon request.

## Acknowledgments

We thank the Friends of the Konrad Lorenz Research Center (Verein der Förderer der Konrad Lorenz Forschungsstelle) and the Cumberland Foundation (Stiftung) for ongoing and long-term support. We thank Josef Hemetsberger for detailed records of greylag goose life history, Julia Rittenschober for data archiving, Benedikt Heger for movement data collection, and Avila Bold, Jonathan Cueva, Marie Fröhlich, Johannes Ploderer, and Jana-Marie Schmincke for collecting data on flight initiation distance. Subgroup departure data were collected by SK during 2020 and 2023, DM during 2021, and JL during 2023. We thank all students and field assistants who helped to record life history and behavioral data of the greylag geese. This work was supported by Austrian Academy of Sciences (W1262-B29 *[10.55776/W1262]*) and Australian Research Council grants (LP210200740) awarded to S.K.

## Author contributions

Conceptualization, S.K., L.K.C., and A.C.K.; methodology, S.K., L.K.C., A.C.K., J.L., D.M., and D.F.; investigation, S.K., L.K.C., A.C.K., J.L., D.M., and D.F.; visualization, L.K.C.; funding acquisition, S.K.; project administration, S.K. and D.F.; supervision, S.K., L.K.C., A.C.K., and D.F.; writing – original draft, S.K., L.K.C., and A.C.K.; writing – review and editing, S.K., L.K.C., A.C.K., J.L., and D.F.

## Declaration of interests

The authors declare no competing interests.

## STAR★Methods

### Key resources table

**Table:** 

REAGENT or RESOURCE	SOURCE	IDENTIFIER
**Deposited data**		

Data	This paper	https://doi.org/10.25365/phaidra.510
R Code	This paper	N/A

**Experimental models: Organisms/strains**		

Greylag goose (*Anser anser*)	Konrad Lorenz Research Center	N/A

**Software and algorithms**		

R Statistical Software	R Project	https://www.r-project.org
Contributed R Packages	Comprehensive R Archive Network (CRAN)	https://cran.r-project.org

**Other**		

GoPro HERO 7 and HERO 9	GoPro Inc.	https://gopro.com/

### Experimental model and study participant details

#### Study population

We studied a free-flying flock of greylag geese (*Anser anser*) that have been habituated to humans across 50 years at the Auingerhof, the former Konrad Lorenz Research Center (KLF: 47°48′49.7412″ N, 13°56′51.72″ E), located in the riverine valley Grünau im Almtal, Upper Austria, Austria. This flock (flock size ranged from 92 to 144 geese over the period of data collection) was introduced to the valley by Konrad Lorenz and colleagues and has been monitored continuously since 1973.^30^^,^^56^ Most geese (98%) are marked with an individually numbered aluminium ring and a unique combination of colorbands. Flock members are non-migratory, as they are supplemented with grain and pellets in six feeding troughs (each 1.5 m × 20 cm) twice per day (morning and late afternoon) throughout the year. During the daily feeding, members of the flock are monitored for presence, social status, pairing status, partner identity, and reproductive success.^56^ All individuals in this study were adults, with 20 females and 97 males ranging from 1 to 23 years old. Therefore, the life history and current social status of each bird within the flock is known. We used these data to calculate individuals’ family size for each month of data collection. Family size included the focal goose, their partner, and any dependent fledglings, i.e., unpaired geese had a family size of one.

The geese are fed at 0800 and 1600 at the Auingerhof, after which time they leave in several smaller subgroups, usually to feeding areas in the adjacent Cumberland Wildpark (CWP; 47°48′37.6704″ N, 13°56′53.9196″ E). In the afternoon, the geese that visit the Auingerhof leave in smaller subgroups to nearby sleeping areas at Lake Alm (47°45′12.1356″ N, 13°57′24.9948″ E), Oberganslbach (47°47′36.762″ N, 13°56′57.2316″ E), or the Cumberland Wildpark.

This study complies with all current Austrian laws and regulations and was supported by Animal Experiment License Number 66.006/0026-WF/V/3b/2014 issued by the Austrian Federal Ministry for Science and Research (EU Standard, equivalent to the Animal Ethics Board). All data collected for this study were obtained using minimally-invasive methods. Birds were habituated to the presence of humans, as the flock has been observed at the Konrad Lorenz Research Center for Behavior and Cognition (KLF) since 1973.

### Method details

#### Sub-group movement group size

During September to November over a four-year period (2020–2023), we systematically recorded group size for 742 leader-follower departure events, which involved 117 individuals that initiated departure calling within their sub-group (mean ± SE events per individual = 6.68 ± 0.84, range 1–41). The researchers observed greylag geese with binoculars at the Auingerhof after the regular morning feeding (0900–1100 local time) or afternoon feeding (1700–1900) and before their departure to other feeding or sleeping areas. Beginning up to half an hour before taking flight, individuals start to walk and position themselves in the direction of departure, with the outcome that their body orientation is parallel to that of other greylag geese, with heads and necks facing the direction of departure.^29^ While this orienting behavior occurs, more individuals start to shake their necks and produce a recruitment call – a repetitive staccato call produced in long bouts of the same syllable.^30^ This is followed by the departure call, a high-amplitude single-element vocalization that is generally produced just before flight departure.^30^ Most of the time (80% of observations in this study), when a departure call is produced, the caller departs shortly thereafter (within seconds to minutes). The departure call is individually distinct.^57^^,^^58^

In this study, we selected sub-groups within the flock for targeted observation to ensure that all flock members were observed at least once every two weeks across a 12-week period. We observed the sub-group as they exhibited pre-departure behaviors and noted the individual that initiated departure calling. We then noted (1) departure group size, the number of geese that departed over the next 2 mins (including the departure caller, their partner, and any fledglings). If no geese took flight within this period, then that individual’s departure group size was recorded as zero; and (2) the identity of the goose that was first to follow a departure caller if and when it took flight, within 2 s of the focal goose’s departure (first-follower ID). We focused on the first follower because this goose can be accurately observed and recorded by a single observer, and also because we considered this goose to be the most highly motivated to follow. In many cases, the first goose to follow was the partner of the departure caller. We did not discriminate between partners and non-partners at the time of data collection, although observations where the partner was the first-follower were later excluded from analysis (see statistical methods below). On average, we recorded 10 departure events per day and randomized the focal geese across days. The variables analyzed were: leader ID, defined as the goose that initiated departure calling; first-follower ID, and group size. The range in group size was 0 (no geese departed within 2 min) to 33 (Figure S3).

#### Personality assays

To measure individual boldness, we conducted human approach trials at the KLF and CWP sites in 2022 and 2023, as per Katsis et al.^23^ For each approach, a target goose was randomly selected while ensuring the goose was stationary, either sitting or standing, and aware of its surroundings (i.e., not resting with its eyes closed). To ensure full visibility of the experimenter, approaches were angled so that the sun was not behind the experimenter. The speed of approach from a starting distance of 10 m was a consistent 0.5 m/s, following a digital metronome played through headphones. Eye contact was kept with the target goose for the full approach duration. For each trial, we measured the goose’s flight initiation distance (FID), which was the distance at which it attempted to flee from the approaching human, either by walking or flying.^59^^,^^60^ Specifically, FID was the distance between the experimenter and the focal goose when the goose took its first step away. Individual differences in FID are significantly repeatable across time both in our study population^23^ and in other goose species.^61^ We recorded the starting posture of the goose, either sitting or standing, as this has been shown to affect FID.^23^ Each goose was only approached once per site per day. To minimise habituation to the experimenter, approach trials were conducted by 11 different people, who were each limited to 7 days of data collection (one experimenter conducted approach trials across two 7-day bouts separated by 13 months). We found no evidence of habituation across trials.^23^ We conducted 762 human approach trials using 111 of the 117 flock members (mean ± SE 6.86 ± 0.31 trials per individual, range 1–18).

To measure individual aggressiveness, we conducted mirror stimulation tests at the KLF site in 2021 and 2023. This type of assay has previously been used to measure aggressiveness across a range of bird species (e.g.,^62^^,^^63^^,^^64^), including greylag geese.^65^ In our study population, responses to mirror stimulation are significantly repeatable and correlate with the number of agonistic interactions that the goose initiates towards flockmates and with its position in the dominance hierarchy.^65^ Geese were first habituated to the presence of four new feeding stations, comprising wooden boards and food trays, during the morning supplementary feeding for three days. On the fourth morning, the wooden boards were fitted with mirrors immediately before feeding. Using local river pebbles, we demarcated 1-m and 2-m zones in front of each mirror. Each trial began when a focal goose entered the 2-m zone. All geese were free to move within and outside of the trial area, and so some geese were tested simultaneously. Each trial lasted for 5 min, regardless of the amount of time the goose spent within the 2-m zone. For each focal goose, we recorded and analysed its minimum distance to the mirror (in cm).^65^ Minimum distance to the mirror does decrease across subsequent trials (Kleindorfer et al. unpublished data). However, this shift is expected to be consistent across individuals, and so individuals that are more aggressive are expected to come closer to the mirror than less aggressive individuals, even in later trials. We conducted 144 mirror stimulation tests for 87 members of the flock (mean ± SE 1.66 ± 0.08 trials per individual, range 1–3). Notably, similar mirror tests are also used in cognitive research to assess self-recognition in some species (e.g.,^66^^,^^67^^,^^68^), although there is no evidence that geese recognise themselves in a mirror. In species that do show self-recognition, individuals tend to pass through four phases of mirror interaction: (i) social responses, (ii) physical inspection (e.g., looking behind the mirror), (iii) repetitive mirror-testing behavior, and (iv) realization of seeing themselves.^69^ Greylag geese widely exhibit the first of these responses, reacting to their mirror-image as though it were a conspecific.^65^ However, a subset of geese also displayed the second behavior response type, inspecting the mirror by looking or walking behind it. This response, which we describe as “mirror inspection”, was scored in 2023 (n = 51) as a binary variable (i.e., the individual did or did not look behind the mirror during the 5-min trial period). In this study, we do not explicitly link mirror inspection with any particular personality trait, as its underlying behavioral mechanisms require further study.

In personality research, neophobia is typically interpreted as an aspect of exploration.^11^ Novel object tests (reviewed in^70^) are a common and repeatable method for measuring neophobia in other species (e.g., common ravens, *Corvus corax*^71^; house sparrows, *Passer domesticus*^72^; orange-winged Amazon parrots, *Amazona amazonica*^73^), including in the barnacle goose.^74^^,^^75^ To measure individual neophobia in greylag geese, we conducted novel object tests at the KLF site in September 2023. First, geese were habituated to the presence of new feeding stations during the morning feeding for three days, following the protocol described above. On the fourth and fifth mornings, we collected the baseline latency for each goose to cross the 1-m mark after entering the 2-m test area (in seconds). A goose was considered to have crossed the 1-m mark if its foot was placed along the 1-m demarcation. If a goose crossed the 2-m boundary but did not cross the 1-m boundary, their latency was recorded as the full test duration (300 s). In the following three days, one novel object was placed directly next to the food tray at two feeding stations, and latency to cross the 1-m boundary was recorded for each goose. The novel objects were (1) small glass jars filled with blue- or red-dyed liquid on day 6; (2) large glass jars filled with green- or yellow-dyed liquid on day 7; and (3) ceramic coffee mugs on day 8. There are a variety of ways that novel objects can be differentiated from each other, including colour, texture, and shape. Varying too many factors can affect neophobic responses and, in previous pilot experiments, we found our geese were incredibly sensitive to novel objects with extreme variation in these attributes. This resulted in uniform avoidance of the feeding area, with geese refusing to return for days. The novel objects used here varied in colour and moderately in shape and were novel enough to provide a distribution of responses in the flock without significant disturbance. No novel objects were reused across the trial days. We found no evidence that geese habituated to the presence of novel objects (Common et al., unpublished data) and so each day is considered as an independent test. Delta latency was calculated using the first baseline latency minus the test latency. We collected 242 delta latency measures for 84 flock members (mean ± SE 2.88 ± 0.04 trials per individual, range 1–3). As with the mirror stimulation test, geese were free to move within and outside of the trial area, and so some geese were tested simultaneously.

### Quantification and statistical analysis

All analyses were conducted in R version 4.1.0.^76^ Univariate linear models (LMs) were calculated using the ‘lm’ function in the package stats v 4.1.0, linear mixed models (LMMs) were calculated using the ‘lmer’ function in the package lme4 v. 1.1.33,^77^ and multivariate generalized linear mixed models (GLMMs) used the function ‘MCMCglmm’ in the package MCMCglmm v. 2.35 Markov chain Monte Carlo generalised linear mixed models^78^. Model assumptions of Gaussian models were assessed using visual assessment of Q–Q plots. We report model effect sizes of LMs and LMMs as estimate ± standard error (SE), using the ‘summary’ function in lme4^77^ and estimated degrees of freedom from lmerTest.^79^ We did not use elimination procedures in any model; therefore, we include all variables of interest and potential sources of variation in the final models regardless of statistical significance. We report χ^2^ and P-values from the ANOVA table of deviance, using Type III χ^2^ tests implemented in the package car^80^ version 3.0–12. We extracted predicted values from LMs and LMMs using the ‘ggpredict’ function in the package ggeffects v. 1.2.3^81^ and created plots using ggplot2 v. 3.3.5.^82^ Data are presented in Dataset S1 and Data S1.

The adjusted repeatability (R) of individuals’ influencer score was calculated using LMMs implemented in the packages lme4^77^ and rptR v. 0.9.22.^83^ To assess individual repeatability,^84^ focal goose ID (influencer role, follower role) was included as the grouping variable and random effect. To fulfill the assumption of normality of residuals, we log-transformed influencer score and used a Gaussian error distribution. Observation month (Julian, continuous), year (categorical), individual hatch year (continuous), and pairing status (categorical: paired or unpaired) were included as fixed effects. The significance of adjusted repeatability was tested using likelihood ratio tests against the null hypothesis that R = 0. We calculated 95% confidence intervals using parametric bootstrapping (1000 iterations). All individuals were included in the dataset for calculating repeatability regardless of number of observations, as all observations contribute to the estimate of total variance.^85^

For the three personality traits that were measured multiple times (boldness, aggressiveness, and neophobia), we calculated adjusted repeatability using the methods described above. For boldness, we used FID (log-transformed) as the dependent variable, with year (categorical), goose position (categorical: sitting or standing), and site (categorical: KLF or CWP) as fixed effects. For aggressiveness, we included year (categorical: 2021 or 2023) as a fixed effect. There were no fixed effects used in the model for neophobia, as this trait was measured at a single site in the same year.

To determine which life history traits predict an individual’s influencer score, we used an LMM with log-transformed influencer score as the response variable. Focal sex (categorical), family size (continuous), hatch year (continuous), and pairing status (categorical) were included as fixed effects. To control for potential differences between observation periods, we also included observation month (continuous) and year (categorical) as fixed effects. Continuous variables were scaled to facilitate the interpretation of effect sizes. Individual ID was included as a random effect.

To test whether the four behavioral traits were associated with influencer score, we used four bivariate (G)LMMs in MCMCglmm, one for each personality trait (boldness, aggressiveness, neophobia) and one for our measure of mirror inspection (looked behind the mirror).^46^^,^^47^ A Gaussian error distribution was used for all factors except for mirror inspection, for which the ordinal distribution was used. Observation month (continuous), family size (continuous), focal sex (categorical), hatch year (continuous), and pairing status (categorical) were included as fixed effects, with individual ID as a random effect. We ran the MCMC for 750,000 iterations (‘nitt’), with 50,000 iteration burn-in and a thinning interval of 175 (see Data S1). Because personality measures were collected on different days to the departure events, estimating the within-individual covariance was inappropriate (see Scenario 4 in Table 2 of^86^) and so we set the within-individual covariance to zero (i.e., rcov = ∼idk(trait):units). The prior used was moderately informative (nu = 1.002) and parameter-expanded,^87^ as trace plots showed no convergence and effective sample sizes were low when using weakly informative (nu = 0.002) and non-parameter-expanded priors. Autocorrelation was assessed using the function ‘autocorr’, ensuring it was <0.1 for each run. Effective sample sizes were >2000 for all models. To confirm model convergence, we ran each model three times with the same prior structure and confirmed that the Gelman Rubin statistic^88^ was approximately 1 for all models. If the 95% credible intervals did not cross zero, the among-individual correlation between the behavioral trait and influencer score was considered statistically significant.

To determine which life history traits influence the number of times a bird was recorded as the first goose to follow, we used an LM with focal sex (categorical), family size (continuous), hatch year (continuous), and pairing status (categorical) as fixed effects. Hatch year was scaled to facilitate interpretation of effect sizes. The residuals of the model were normal and, therefore, the data were not transformed. When calculating the total number of times a goose was first-to-follow a departure caller, we excluded events in which the goose was first to follow its partner (n = 280). Because greylag goose pairs usually take flight together, we expected partners to be frequent first-followers regardless of their own personality phenotype, which would obscure any additional patterns if there were any. There were no recorded observations where a fledgling was the first to follow.

To test whether the three personality traits (boldness, aggressiveness, and neophobia) were associated with the number of times a goose was first to follow, we used three bivariate LMMs in MCMCglmm, following methods described above (see Data S1). First-follower count was log-transformed to fulfill the assumption of normality of residuals and facilitate model convergence. Focal sex (categorical) was included as a fixed effect and individual ID was included as a random effect. Including family size, hatch year, and pairing status as factors caused convergence issues and had low effective sample size; therefore, these variables were omitted from the model. We ran the MCMC for 1,000,000 iterations (‘nitt’), with 50,000 iteration burn-in and a thinning interval of 300. We increased the number of iterations and burn-in to facilitate model mixing. The prior used was parameter-expanded and moderately informative (nu = 1.002), with variation in first-follower count fixed to one as there was only one measure per individual. Model convergence was assessed using the diagnostic methods described above. The association between mirror inspection (whether the individual looked behind the mirror) and being the first to follow was tested using an LM with sex as a fixed effect.

## Supplemental information

Document S1. Figures S1–S3 and Tables S1–S3

Dataset S1. Dataset used in analysis

Data S1. R code used for analysis
