# Supplementary material for: Personality predicts collective behavior in greylag geese: Influencers are bold and followers are exploratory
Source: iScience. 2025 Jul 22;28(8):113170. doi: 10.1016/j.isci.2025.113170 (PMC12355418; doi:10.1016/j.isci.2025.113170)
Supplement: Document S1. Figures S1–S3 and Tables S1–S3 [file mmc1.pdf]

iScience, Volume *28*

Supplemental information

Personality predicts collective behavior in greylag geese: Influencers are bold and followers are exploratory

Sonia Kleindorfer, Andrew C. Katsis, Didone Frigerio, Jonas Lesigang, Dina Mostafa, and Lauren K. Common

Supplemental Information

Document S1. Figures S1–S3 and Tables S1–S3.

Video file examples of subgroup departure events.

https://youtu.be/6e4fUUwCZrE

https://youtu.be/kDupt5nzOoY

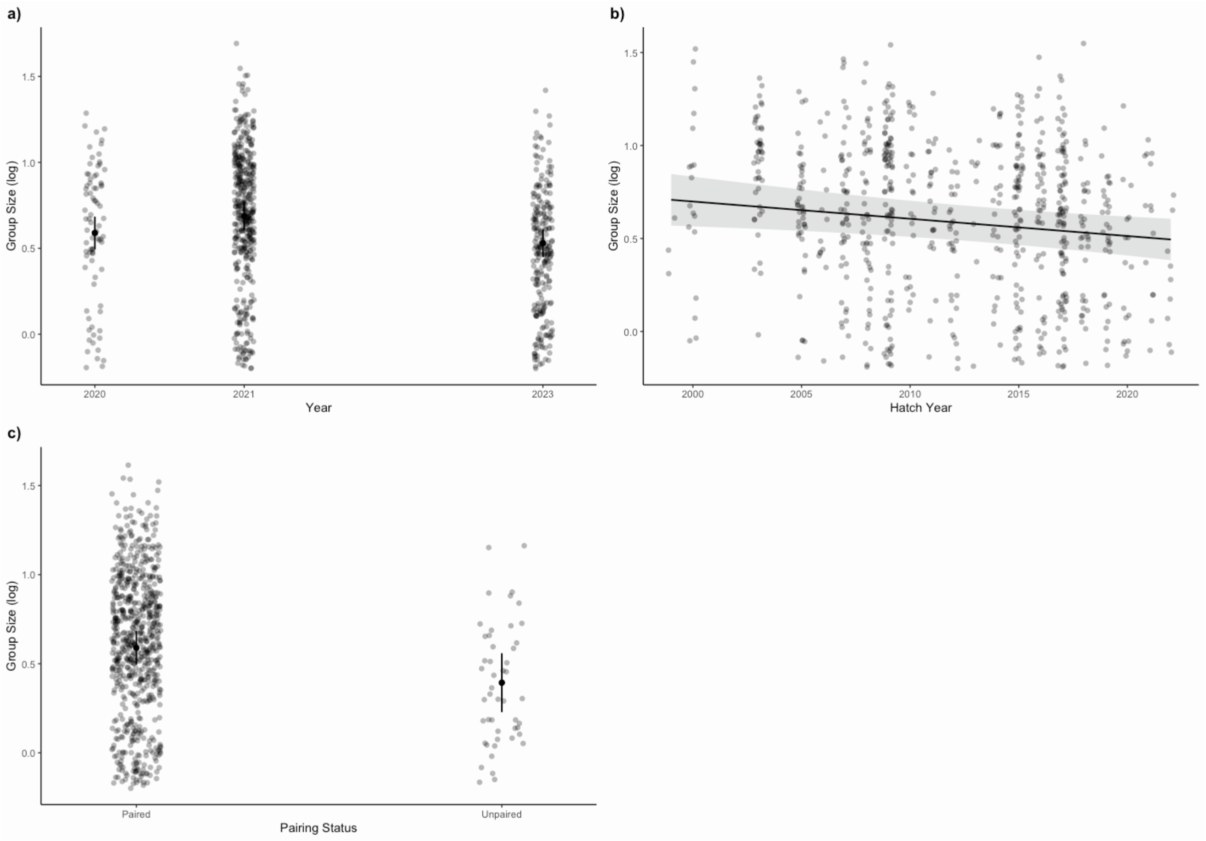

Figure S1. Factors associated with group size during collective movement, related to Table 1. Effects of a) year, b) focal hatch year, and c) focal pairing status on group size after influencer departure in greylag geese (N = 742 observations for 117 individuals). Raw data are presented as grey circles with jitter for clarity. Black bars in a, c) and grey ribbons in b) represent 95% confidence intervals around estimated marginal means.

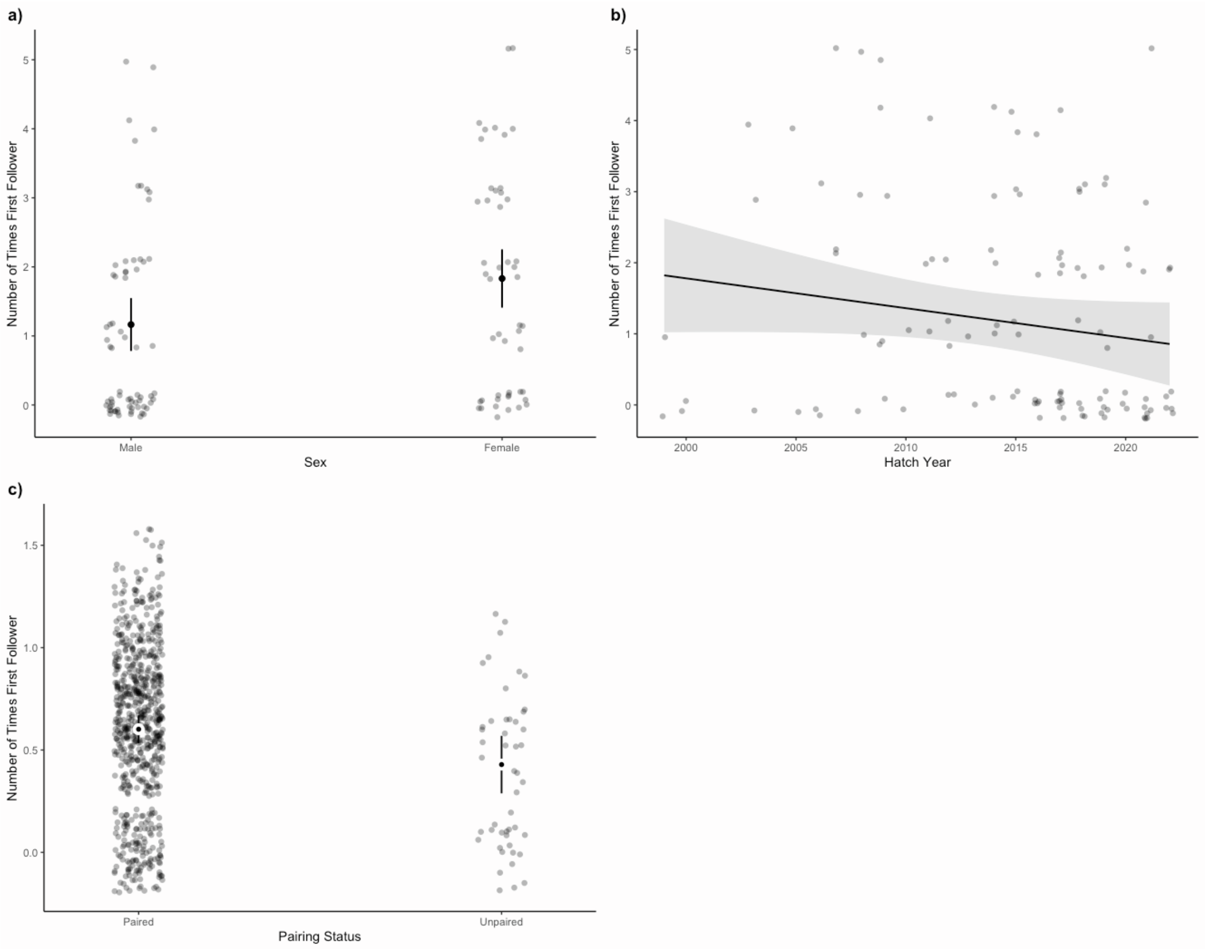

Figure S2. Factors associated with the probability of being the first to follow a departing goose, related to Table 2. Effects of a) focal sex, b) hatch year and c) pairing status on the number of times a goose was recorded as being the first to follow throughout the study period (2020–2023, N = 117 individuals). Raw data are presented as grey circles with jitter for clarity. Grey ribbons in b) and black bars in a,c) represent 95% confidence intervals around estimated marginal means.

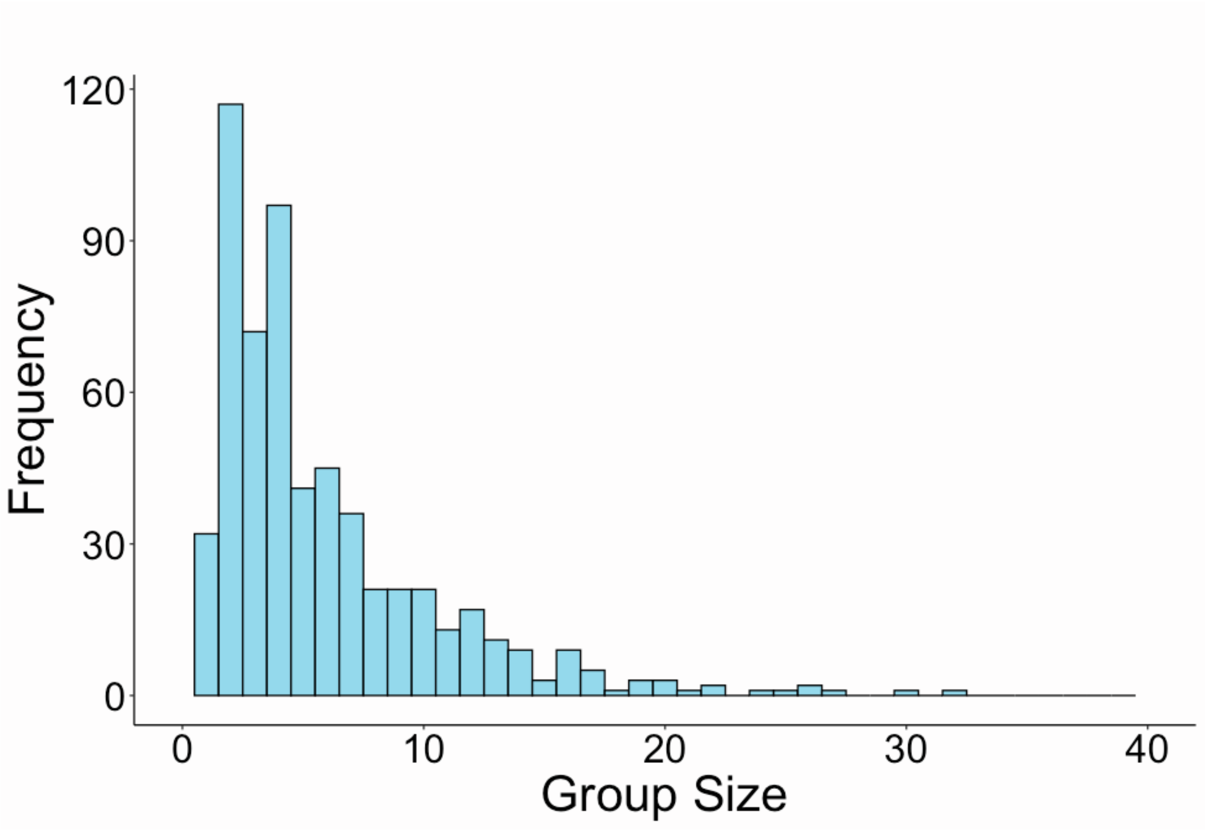

Figure S3. The distribution of group size during collective movement, related to Table 1. The range and frequency of group sizes during departure events in greylag geese.

Table S1. Personality traits associated with group size during collective movement, related to Figures 1 and 2. Output from multivariate (generalised) linear mixed models showing variances (diagonals), covariances (below diagonals), and correlations (above diagonals) between departure group size (log-transformed) after departure calling and three different personality traits.

a) Boldness

	Departure group size	Boldness
Departure group size	0.011 (0.001, 0.021)	-0.786 (-0.997, -0.524)
Boldness	-0.008 (-0.013, -0.003)	0.007 (0.003, 0.011)

b) Aggressiveness

	Departure group size	Aggressiveness
Departure group size	0.011 (< 0.001, 0.021)	0.179 (-0.530, 0.815)
Aggressiveness	0.658 (-1.424, 3.158)	1248 (0.014, 2410)

c) Neophobia

	Departure group size	Neophobia
Departure group size	0.010 (< 0.001, 0.020)	0.050 (-0.571, 0.666)
Neophobia	0.696 (-6.025, 7.491)	14,810 (8,085, 21,600)

d) Mirror inspection

	Departure group size	Inspection
Departure group size	0.011 (0.002, 0.021)	< 0.001 (-0.064, 0.057)
Mirror inspection	0.001 (-0.170, 0.168)	627.5 (< 0.001, 2459)

Personality was measured across three traits: a) boldness (flight initiation distance, log-transformed), b) aggressiveness (minimum distance to mirror during a mirror stimulation test), and c) neophobia (difference in latency from 2 m to 1 m between baseline and treatment phases of a novel object test) and d) mirror inspection (ordinal distribution, looked behind the mirror during a mirror stimulation test). Lower and upper bounds of 95% credible intervals are shown in parentheses.

Table S2. Personality traits associated with being the first to follow a departing goose, related to Figure 3. Output from multivariate LMMs showing variances (diagonals), covariances (below diagonals), and correlations (above diagonals) between the number of times a goose was recorded as the first to follow (log-transformed) and three different personality traits.

a) Boldness

	First to follow	Boldness
First to follow	0.076 (0.056, 0.097)	-0.033 (-0.162, 0.227)
Boldness	0.001 (-0.006, 0.008)	0.019 (0.015, 0.025)

b) Aggressiveness

	First to follow	Aggressiveness
First to follow	0.077 (0.060, 0.099)	-0.078 (-0.268, 0.116)
Aggressiveness	-1.387 (-5.095, 2.007)	4046 (3,110, 5,114)

c) Neophobia

	First to follow	Neophobia
First to follow	0.077 (0.058, 0.098)	-0.196 (-0.373, -0.007)
Neophobia	-7.916 (-16.240, -0.227)	20,880 (15,770, 26,570)

Personality was measured across three traits: a) boldness (flight initiation distance, log-transformed), b) aggressiveness (minimum distance to mirror during a mirror stimulation test), and c) neophobia (difference in latency from 2 m to 1 m between baseline and treatment phases of a novel object test). Lower and upper bounds of 95% credible intervals are shown in parentheses.

Table S3. Mirror inspection behavior associated with being the first to follow a departing goose, related to Figure 4. Output from a linear model testing the effect of mirror inspection and focal sex on the number of times a goose was recorded as being the first to follow. First-to-follow data were collected throughout the study period (2020–2023, N = 51 individuals). Mirror inspection is defined as the individual looking behind the mirror during the mirror stimulation test.

	**Estimate**	**SE**	**T**	**SumSq**	**F**	**P**
Intercept	2.143	0.33	6.585			
Inspection	1.430	0.51	2.829	15.13	8.00	**0.007**
Focal Sex[a]	-1.192	0.40	-2.991	16.92	8.95	**0.004**

[a] Female used as reference category. Bold values indicate statistical significance ($P < 0.05$).
