# Supplementary material for: Personality predicts collective behavior in greylag geese: Influencers are bold and followers are exploratory
Source: iScience. 2025 Jul 22;28(8):113170. doi: 10.1016/j.isci.2025.113170 (PMC12355418; doi:10.1016/j.isci.2025.113170)
Supplement: Data S1. R code used for analysis [file mmc3.docx]

##### Personality predicts collective behavior in greylag geese: Influencers are bold and followers are exploratory #####

### Sonia Kleindorfer, Andrew C. Katsis, Didone Frigerio, Jonas Lesigang, Dina Mostafa, Lauren K. Common ###

## iScience 2025 ##

library(readxl)

library(MCMCglmm)

library(car)

library(effects)

library(lmerTest)

library(rptR)

library(ggplot2)

library(ggeffects)

library(tidyverse)

library(ggpubr)

library(MASS)

library(dplyr)

# Import Data sheet of MS Kleindorfer et al. Data as 'influencer'

influencer <- Data_S1_Kleindorfer_et_al

# Log transform variables #

influencer$log_Group_Size <- log10(influencer$Group_Size+1)

influencer$log_Bold_FID <- log10(influencer$Bold_FID+1)

influencer$log.Follower <- log10(influencer$Number_Times_Follower+1)

# Set as categorical variable #

influencer$Look_Behind_Mirror <- as.factor(influencer$Look_Behind_Mirror)

influencer$Year <- as.factor(influencer$Year)

influencer$Year_FID <- as.factor(influencer$Year_FID)

influencer$Aggr_Year <- as.factor(influencer$Aggr_Year)

##### Repeatability Analyses #####

### Group Size ###

gs.rpt <- rpt(log_Group_Size ~ scale(Month) + Year + scale(Hatch_Year) + Family_Size + Focal_Pairing_Status + (1|FocalID), grname = "FocalID",

data = influencer, datatype = "Gaussian", nboot = 1000, npermut = 0)

summary(gs.rpt)

### Boldness ###

bold.rpt <- rpt(log_Bold_FID ~ Year_FID + Position_FID + Site_FID+ (1|FocalID), grname = "FocalID",

data = influencer, datatype = "Gaussian", nboot = 1000, npermut = 0)

summary(bold.rpt)

### Aggressiveness ###

aggr.rpt <- rpt(Aggr_Min_Dist ~ Aggr_Year + (1|FocalID), grname = "FocalID",

data = influencer, datatype = "Gaussian", nboot = 1000, npermut = 0)

summary(aggr.rpt)

### Neophobia ###

neo.rpt <- rpt(Neo_Delta_Latency ~ (1|FocalID), grname = "FocalID",

data = influencer, datatype = "Gaussian", nboot = 1000, npermut = 0)

summary(neo.rpt)

##### Effect of sex, age and pairing status on group size #####

life.gs.lm <- lmer(log_Group_Size ~ scale(Month) + Year + Focal_Sex + scale(Family_Size) + scale(Hatch_Year) + Focal_Pairing_Status

+ (1|FocalID), data = influencer)

##### Table 1 #####

# Table columns 1-5

summary(life.gs.lm)

# Table columns 6-7

Anova(life.gs.lm)

##### Figure S1 #####

life.gs.pred.yr <- ggpredict(life.gs.lm, terms = c("Year"))

p1 <- plot(life.gs.pred.yr, add.data = TRUE, jitter = TRUE) +

labs(x = "Year", y = "Group Size (log)", title = "") +

theme_ggeffects(base_size = 16) +

theme_classic()

life.gs.pred.age <- ggpredict(life.gs.lm, terms = c("Hatch_Year"))

p2 <- plot(life.gs.pred.age, add.data = TRUE, jitter = TRUE) +

labs(x = "Hatch Year", y = "Group Size (log)", title = "") +

theme_ggeffects(base_size = 16) +

theme_classic()

life.gs.pred.pair <- ggpredict(life.gs.lm, terms = c("Focal_Pairing_Status"))

p3 <- plot(life.gs.pred.pair, add.data = TRUE, jitter = TRUE) +

labs(x = "Pairing Status", y = "Group Size (log)", title = "") +

theme_ggeffects(base_size = 16) +

theme_classic()

FigureS1 <- ggarrange(p1, p2, p3, nrow=2, ncol=2, labels = c("a)", "b)", "c)"))

FigureS1

##### Bivariate Models - Influencers and Personality #####

# Priors #

prior1 = list(R = list(V = diag(2), nu = 1.002),

G = list(G1 = list(V = diag(2), nu = 2,

alpha.mu = rep(0,2),

alpha.V = diag(25^2,2,2))))

prior_lbm = list(R = list(V = diag(1, 2), nu = 1.002),

G = list(G1 = list(V = diag(2), nu = 1000,

alpha.mu = rep(0,2),

alpha.V = diag(25^2,2,2))))

##### Table S1 #####

# Extracted from summary(), mean() and HPDinterval() of the four models below

### Influencer and Boldness ###

mcmc.influencer.boldness <- MCMCglmm(cbind(log_Group_Size, log_Bold_FID) ~ trait

+ at.level(trait,1):scale(Month) + at.level(trait,1):scale(Family_Size)

+ trait:Focal_Sex + trait:scale(Hatch_Year) + trait:Focal_Pairing_Status,

random =~ us(trait):FocalID,

rcov =~ idh(trait):units,

family = c("gaussian", "gaussian"),

prior = prior1,

nitt = 750000,

burnin = 50000,

thin = 175,

verbose = TRUE,

pr = TRUE,

data = as.data.frame(influencer))

summary(mcmc.influencer.boldness)

influencer_boldness <- mcmc.influencer.boldness$VCV[,"traitlog_Bold_FID:traitlog_Group_Size.FocalID"]/

(sqrt(mcmc.influencer.boldness$VCV[,"traitlog_Bold_FID:traitlog_Bold_FID.FocalID"])*

sqrt(mcmc.influencer.boldness$VCV[,"traitlog_Group_Size:traitlog_Group_Size.FocalID"]))

mean(influencer_boldness)

HPDinterval(influencer_boldness)

#credible intervals DO NOT overlap zero

# Model diagnostics

plot.MCMCglmm(mcmc.influencer.boldness)

plot(mcmc.influencer.boldness$Sol)

plot(mcmc.influencer.boldness$VCV)

autocorr(mcmc.influencer.boldness$VCV)[,,"traitlog_Group_Size:traitlog_Group_Size.FocalID"][3,4]

autocorr(mcmc.influencer.boldness$VCV)[,,"traitlog_Bold_FID:traitlog_Bold_FID.FocalID"][3,4]

# Alternative model run #1

mcmc.influencer.boldness1 <- MCMCglmm(cbind(log_Group_Size, log_Bold_FID) ~ trait

+ at.level(trait,1):scale(Month) + at.level(trait,1):scale(Family_Size)

+ trait:Focal_Sex + trait:scale(Hatch_Year) + trait:Focal_Pairing_Status,

random =~ us(trait):FocalID,

rcov =~ idh(trait):units,

family = c("gaussian", "gaussian"),

prior = prior1,

nitt = 750000,

burnin = 50000,

thin = 175,

verbose = TRUE,

pr = TRUE,

data = as.data.frame(influencer))

# Alternative model run #2

mcmc.influencer.boldness2 <- MCMCglmm(cbind(log_Group_Size, log_Bold_FID) ~ trait

+ at.level(trait,1):scale(Month) + at.level(trait,1):scale(Family_Size)

+ trait:Focal_Sex + trait:scale(Hatch_Year) + trait:Focal_Pairing_Status,

random =~ us(trait):FocalID,

rcov =~ idh(trait):units,

family = c("gaussian", "gaussian"),

prior = prior1,

nitt = 750000,

burnin = 50000,

thin = 175,

verbose = TRUE,

pr = TRUE,

data = as.data.frame(influencer))

chainList <- mcmc.list(mcmc.influencer.boldness$Sol,mcmc.influencer.boldness1$Sol, mcmc.influencer.boldness2$Sol)

plot(chainList)

gelman.diag(chainList)

gelman.plot(chainList)

### Influencer and Aggressiveness ###

mcmc.influencer.aggr <- MCMCglmm(cbind(log_Group_Size, Aggr_Min_Dist) ~ trait

+ at.level(trait,1):scale(Month) + at.level(trait,1):scale(Family_Size)

+ trait:Focal_Sex + trait:scale(Hatch_Year) + trait:Focal_Pairing_Status,

random =~ us(trait):FocalID,

rcov =~ idh(trait):units,

family = c("gaussian", "gaussian"),

prior = prior1,

nitt = 750000,

burnin = 50000,

thin = 175,

verbose = TRUE,

pr = TRUE,

data = as.data.frame(influencer))

summary(mcmc.influencer.aggr)

influencer_aggr <- mcmc.influencer.aggr$VCV[,"traitAggr_Min_Dist:traitlog_Group_Size.FocalID"]/

(sqrt(mcmc.influencer.aggr$VCV[,"traitAggr_Min_Dist:traitAggr_Min_Dist.FocalID"])*

sqrt(mcmc.influencer.aggr$VCV[,"traitlog_Group_Size:traitlog_Group_Size.FocalID"]))

mean(influencer_aggr)

HPDinterval(influencer_aggr)

#credible intervals overlap zero

# Model diagnostics

plot.MCMCglmm(mcmc.influencer.aggr)

plot(mcmc.influencer.aggr$Sol)

plot(mcmc.influencer.aggr$VCV)

autocorr(mcmc.influencer.aggr$VCV)[,,"traitlog_Group_Size:traitlog_Group_Size.FocalID"][3,4]

autocorr(mcmc.influencer.aggr$VCV)[,,"traitAggr_Min_Dist:traitAggr_Min_Dist.FocalID"][3,4]

# Alternative model run #1

mcmc.influencer.aggr1 <- MCMCglmm(cbind(log_Group_Size, Aggr_Min_Dist) ~ trait

+ at.level(trait,1):scale(Month) + at.level(trait,1):scale(Family_Size)

+ trait:Focal_Sex + trait:scale(Hatch_Year) + trait:Focal_Pairing_Status,

random =~ us(trait):FocalID,

rcov =~ idh(trait):units,

family = c("gaussian", "gaussian"),

prior = prior1,

nitt = 750000,

burnin = 50000,

thin = 175,

verbose = TRUE,

pr = TRUE,

data = as.data.frame(influencer))

# Alternative model run #2

mcmc.influencer.aggr2 <- MCMCglmm(cbind(log_Group_Size, Aggr_Min_Dist) ~ trait

+ at.level(trait,1):scale(Month) + at.level(trait,1):scale(Family_Size)

+ trait:Focal_Sex + trait:scale(Hatch_Year) + trait:Focal_Pairing_Status,

random =~ us(trait):FocalID,

rcov =~ idh(trait):units,

family = c("gaussian", "gaussian"),

prior = prior1,

nitt = 750000,

burnin = 50000,

thin = 175,

verbose = TRUE,

pr = TRUE,

data = as.data.frame(influencer))

chainList <- mcmc.list(mcmc.influencer.aggr$Sol,mcmc.influencer.aggr1$Sol, mcmc.influencer.aggr2$Sol)

plot(chainList)

gelman.diag(chainList)

gelman.plot(chainList)

### Influencer and Neophobia ###

mcmc.influencer.neo <- MCMCglmm(cbind(log_Group_Size, Neo_Delta_Latency) ~ trait

+ at.level(trait,1):scale(Month) + at.level(trait,1):scale(Family_Size)

+ trait:Focal_Sex + trait:scale(Hatch_Year) + trait:Focal_Pairing_Status,

random =~ us(trait):FocalID,

rcov =~ idh(trait):units,

family = c("gaussian", "gaussian"),

prior = prior1,

nitt = 750000,

burnin = 50000,

thin = 175,

verbose = TRUE,

pr = TRUE,

data = as.data.frame(influencer))

summary(mcmc.influencer.neo)

influencer_neo <- mcmc.influencer.neo$VCV[,"traitNeo_Delta_Latency:traitlog_Group_Size.FocalID"]/

(sqrt(mcmc.influencer.neo$VCV[,"traitNeo_Delta_Latency:traitNeo_Delta_Latency.FocalID"])*

sqrt(mcmc.influencer.neo$VCV[,"traitlog_Group_Size:traitlog_Group_Size.FocalID"]))

mean(influencer_neo)

HPDinterval(influencer_neo)

#credible intervals overlap zero

# Model diagnostics

plot.MCMCglmm(mcmc.influencer.neo)

plot(mcmc.influencer.neo$Sol)

plot(mcmc.influencer.neo$VCV)

autocorr(mcmc.influencer.neo$VCV)[,,"traitlog_Group_Size:traitlog_Group_Size.FocalID"][3,4]

autocorr(mcmc.influencer.neo$VCV)[,,"traitNeo_Delta_Latency:traitNeo_Delta_Latency.FocalID"][3,4]

# Alternative model run #1

mcmc.influencer.neo1 <- MCMCglmm(cbind(log_Group_Size, Neo_Delta_Latency) ~ trait

+ at.level(trait,1):scale(Month) + at.level(trait,1):scale(Family_Size)

+ trait:Focal_Sex + trait:scale(Hatch_Year) + trait:Focal_Pairing_Status,

random =~ us(trait):FocalID,

rcov =~ idh(trait):units,

family = c("gaussian", "gaussian"),

prior = prior1,

nitt = 750000,

burnin = 50000,

thin = 175,

verbose = TRUE,

pr = TRUE,

data = as.data.frame(influencer))

# Alternative model run #2

mcmc.influencer.neo2 <- MCMCglmm(cbind(log_Group_Size, Neo_Delta_Latency) ~ trait

+ at.level(trait,1):scale(Month) + at.level(trait,1):scale(Family_Size)

+ trait:Focal_Sex + trait:scale(Hatch_Year) + trait:Focal_Pairing_Status,

random =~ us(trait):FocalID,

rcov =~ idh(trait):units,

family = c("gaussian", "gaussian"),

prior = prior1,

nitt = 750000,

burnin = 50000,

thin = 175,

verbose = TRUE,

pr = TRUE,

data = as.data.frame(influencer))

chainList <- mcmc.list(mcmc.influencer.neo$Sol,mcmc.influencer.neo1$Sol, mcmc.influencer.neo2$Sol)

plot(chainList)

gelman.diag(chainList)

gelman.plot(chainList)

##### Mirror Exploration #####

mcmc.influencer.lbm <- MCMCglmm(cbind(log_Group_Size, Look_Behind_Mirror) ~ trait

+ at.level(trait,1):scale(Month) + at.level(trait,1):scale(Family_Size)

+ trait:Focal_Sex + trait:scale(Hatch_Year) + trait:Focal_Pairing_Status,

random =~ us(trait):FocalID,

rcov =~ idh(trait):units,

family = c("gaussian", "ordinal"),

prior = prior_lbm,

nitt = 750000,

burnin = 50000,

thin = 175,

verbose = TRUE,

pr = TRUE,

data = as.data.frame(influencer))

summary(mcmc.influencer.lbm)

influencer_lbm <- mcmc.influencer.lbm$VCV[,"traitLook_Behind_Mirror:traitlog_Group_Size.FocalID"]/

(sqrt(mcmc.influencer.lbm$VCV[,"traitLook_Behind_Mirror:traitLook_Behind_Mirror.FocalID"])*

sqrt(mcmc.influencer.lbm$VCV[,"traitlog_Group_Size:traitlog_Group_Size.FocalID"]))

mean(influencer_lbm)

HPDinterval(influencer_lbm)

#credible intervals overlap zero

# Model diagnostics

plot.MCMCglmm(mcmc.influencer.lbm)

plot(mcmc.influencer.lbm$Sol)

plot(mcmc.influencer.lbm$VCV)

autocorr(mcmc.influencer.lbm$VCV)[,,"traitlog_Group_Size:traitlog_Group_Size.FocalID"][3,4]

autocorr(mcmc.influencer.lbm$VCV)[,,"traitLook_Behind_Mirror:traitLook_Behind_Mirror.FocalID"][3,4]

# Alternative model run #1

mcmc.influencer.lbm1 <- MCMCglmm(cbind(log_Group_Size, Look_Behind_Mirror) ~ trait

+ at.level(trait,1):scale(Month) + at.level(trait,1):scale(Family_Size)

+ trait:Focal_Sex + trait:scale(Hatch_Year) + trait:Focal_Pairing_Status,

random =~ us(trait):FocalID,

rcov =~ idh(trait):units,

family = c("gaussian", "ordinal"),

prior = prior_lbm,

nitt = 750000,

burnin = 50000,

thin = 175,

verbose = TRUE,

pr = TRUE,

data = as.data.frame(influencer))

# Alternative model run #2

mcmc.influencer.lbm2 <- MCMCglmm(cbind(log_Group_Size, Look_Behind_Mirror) ~ trait

+ at.level(trait,1):scale(Month) + at.level(trait,1):scale(Family_Size)

+ trait:Focal_Sex + trait:scale(Hatch_Year) + trait:Focal_Pairing_Status,

random =~ us(trait):FocalID,

rcov =~ idh(trait):units,

family = c("gaussian", "ordinal"),

prior = prior_lbm,

nitt = 750000,

burnin = 50000,

thin = 175,

verbose = TRUE,

pr = TRUE,

data = as.data.frame(influencer))

chainList <- mcmc.list(mcmc.influencer.lbm$Sol,mcmc.influencer.lbm1$Sol, mcmc.influencer.lbm2$Sol)

plot(chainList)

gelman.diag(chainList)

gelman.plot(chainList)

##### Figure 2 #####

# Boldness

boldness_influencer_cors <- tibble(Traits = c("log_Bold_FID, log_Group_Size"),

Estimate = mean(influencer_boldness),

Lower = c(HPDinterval(influencer_boldness)[,"lower"]),

Upper = c(HPDinterval(influencer_boldness)[,"upper"]))

bold_influencer_plot <- ggplot(boldness_influencer_cors, aes(x = Traits, y = Estimate)) +

geom_pointrange(aes(ymin = Lower, ymax = Upper)) +

geom_hline(yintercept = 0, linetype = "dotted", alpha = 0.3) +

scale_x_discrete(labels = element_blank()) +

theme_classic() +

theme(panel.border = element_rect(colour = "black", fill=NA), panel.background = element_rect(fill="white"), panel.grid.major = element_blank(),

panel.grid.minor = element_blank(), axis.line = element_line(colour = "black"),

axis.title.x = element_text(colour="black", size=14),

axis.title.y = element_text(colour="black", size=14, angle = 0, vjust = 0.5)) +

labs(x = " Boldness",

y = "") +

ylim(-1,1) + coord_flip()

bold_influencer_plot

# Aggressiveness

aggr_influencer_cors<- tibble(Traits = c("Aggr_Min_Dist, log_Group_Size"),

Estimate = mean(influencer_aggr),

Lower = c(HPDinterval(influencer_aggr)[,"lower"]),

Upper = c(HPDinterval(influencer_aggr)[,"upper"]))

aggr_influencer_plot <- ggplot(aggr_influencer_cors, aes(x = Traits, y = Estimate)) +

geom_pointrange(aes(ymin = Lower, ymax = Upper)) +

geom_hline(yintercept = 0, linetype = "dotted", alpha = 0.3) +

scale_x_discrete(labels = element_blank()) +

theme_classic() +

theme(panel.border = element_rect(colour = "black", fill=NA), panel.background = element_rect(fill="white"), panel.grid.major = element_blank(),

panel.grid.minor = element_blank(), axis.line = element_line(colour = "black"),

axis.title.x = element_text(colour="black", size=14),

axis.title.y = element_text(colour="black", size=14, angle = 0, vjust = 0.5)) +

labs(x = "Aggressiveness",

y = "") +

ylim(-1,1) + coord_flip()

aggr_influencer_plot

# Neophobia

neo_influencer_cors<- tibble(Traits = c("Neo_Delta_Latency, log_Group_Size"),

Estimate = mean(influencer_neo),

Lower = c(HPDinterval(influencer_neo)[,"lower"]),

Upper = c(HPDinterval(influencer_neo)[,"upper"]))

neo_influencer_plot <- ggplot(neo_influencer_cors, aes(x = Traits, y = Estimate)) + coord_flip() +

geom_pointrange(aes(ymin = Lower, ymax = Upper)) +

geom_hline(yintercept = 0, linetype = "dotted", alpha = 0.3) +

scale_x_discrete(labels = element_blank()) +

theme_classic() +

theme(panel.border = element_rect(colour = "black", fill=NA), panel.background = element_rect(fill="white"), panel.grid.major = element_blank(),

panel.grid.minor = element_blank(), axis.line = element_line(colour = "black"),

axis.title.x = element_text(colour="black", size=14),

axis.title.y = element_text(colour="black", size=14, angle = 0, vjust = 0.5)) +

labs(x = " Neophobia",

y = "") +

ylim(-1,1)

neo_influencer_plot

# Exploration

exp_influencer_cors<- tibble(Traits = c("Look_Behind_Mirror, log_Group_Size"),

Estimate = mean(influencer_lbm),

Lower = c(HPDinterval(influencer_lbm)[,"lower"]),

Upper = c(HPDinterval(influencer_lbm)[,"upper"]))

exp_influencer_plot <- ggplot(exp_influencer_cors, aes(x = Traits, y = Estimate)) + coord_flip() +

geom_pointrange(aes(ymin = Lower, ymax = Upper)) +

geom_hline(yintercept = 0, linetype = "dotted", alpha = 0.3) +

scale_x_discrete(labels = element_blank()) +

theme_classic() +

theme(panel.border = element_rect(colour = "black", fill=NA), panel.background = element_rect(fill="white"), panel.grid.major = element_blank(),

panel.grid.minor = element_blank(), axis.line = element_line(colour = "black"),

axis.title.x = element_text(colour="black", size=14),

axis.title.y = element_text(colour="black", size=14, angle = 0, vjust = 0.5)) +

labs(x = " Exploration",

y = "Correlation (Estimate +/- 95% CIs)") +

ylim(-1,1)

exp_influencer_plot

# Arrange

Figure1 <- ggarrange(bold_influencer_plot, aggr_influencer_plot, neo_influencer_plot, exp_influencer_plot, nrow=4, ncol=1, labels = c("a)", "b)", "c)", "d)"))

Figure1

##### Figure 1 #####

boldness_coefs <- tibble(Trait = attr(colMeans(mcmc.influencer.boldness$Sol), "names"),

Value = colMeans(mcmc.influencer.boldness$Sol)) %>%

separate(Trait, c("Trait","Type","FocalID"), sep = "\\.", fill = "right") %>%

filter(Type == "FocalID") %>%

filter(Trait %in% c("traitlog_Group_Size", "traitlog_Bold_FID")) %>%

select(-Type) %>%

spread(Trait, Value)

print(boldness_coefs, n = 117)

# Calculate slope

B_fit_slope <- mcmc.influencer.boldness$VCV[,"traitlog_Group_Size:traitlog_Bold_FID.FocalID"]/

mcmc.influencer.boldness$VCV[,"traitlog_Bold_FID:traitlog_Bold_FID.FocalID"]

# Plot scatterplot of BLUPs

ggplot(boldness_coefs, aes(x = traitlog_Bold_FID, y = traitlog_Group_Size, group = FocalID)) +

geom_point(fill=NA, size=4, shape=1, stroke=0.8) + geom_abline(intercept = 0, slope = mean(B_fit_slope))+

theme_classic()+

theme(panel.border = element_rect(colour = "black", fill=NA, linewidth=0.8), panel.background = element_rect(fill="white"), panel.grid.major = element_blank(),

panel.grid.minor = element_blank(), axis.line = element_line(colour = "black"),

axis.title.x = element_text(colour="black", size=18),

axis.title.y = element_text(colour="black", size=18)) +

labs(x = "Flight Initiation Distance\n(BLUP)", y = "Group size\n(BLUP)") +

theme(axis.text.x= element_text(colour="black", size=14),

axis.text.y= element_text(colour="black", size=14), legend.title=element_text(size=14),

legend.text=element_text(size=14), legend.key.size = unit(3,"line"))

##### Effect of sex, age and pairing status on number of times recorded as first follower #####

life.fol.lm1 <- lm(Number_Times_Follower ~ Focal_Sex + scale(Family_Size) + scale(Hatch_Year) + Focal_Pairing_Status, data = influencer)

##### Table 2 #####

# Table columns 1-5

# Table columns 6-7

summary(life.fol.lm1)

Anova(life.fol.lm1)

##### Figure S2 #####

life.fol.pred.sex <- ggpredict(life.fol.lm1, terms = c("Focal_Sex"))

p2.1 <- plot(life.fol.pred.sex, add.data = TRUE, jitter = TRUE) +

labs(x = "Sex", y = "Number of Times First Follower", title = "") +

theme_ggeffects(base_size = 16) +

theme_classic()

life.fol.pred.age <- ggpredict(life.fol.lm1, terms = c("Hatch_Year"))

p2.2 <- plot(life.fol.pred.age, add.data = TRUE, jitter = TRUE) +

labs(x = "Hatch Year", y = "Number of Times First Follower", title = "") +

theme_ggeffects(base_size = 16) +

theme_classic()

life.fol.pred.pair <- ggpredict(life.fol.lm1, terms = c("Focal_Pairing_Status"))

p2.3 <- plot(life.gs.pred.pair, add.data = TRUE, jitter = TRUE) +

labs(x = "Pairing Status", y = "Number of Times First Follower", title = "") +

theme_ggeffects(base_size = 16) +

theme_classic()

FigureS2 <- ggarrange(p2.1, p2.2, p2.3, nrow=2, ncol=2, labels = c("a)", "b)", "c)"))

FigureS2

##### Mirror Exploration and First Follower #####

lbm.fol.lm1 <- lm(Number_Times_Follower ~ Look_Behind_Mirror + Focal_Sex, data = influencer)

##### Table S3 #####

summary(lbm.fol.lm1)

Anova(lbm.fol.lm1)

##### Figure 4 #####

lbm.fol.pred <- ggpredict(lbm.fol.lm1, terms = c("Look_Behind_Mirror"))

plot(lbm.fol.pred, add.data = TRUE, jitter = TRUE) +

labs(x = "Looked Behind Mirror", y = "Number of Times First to Follow", title = "") +

theme_ggeffects(base_size = 16) +

theme_classic(base_size = 16)

##### Bivariate Models - Number of times as first follower and personality #####

##### Table S2 #####

# Extracted from summary(), mean() and HPDinterval() of the four models below

# Priors #

prior_follow = list(R = list(V = diag(0.0001, 2), nu = 1.002, fix = 1),

G = list(G1 = list(V = diag(2), nu = 2,

alpha.mu = rep(0,2),

alpha.V = diag(25^2,2,2))))

### Followers and Boldness ###

mcmc.follower.boldness <- MCMCglmm(cbind(log.Follower, log_Bold_FID) ~ trait

+ trait:Focal_Sex,

random =~ us(trait):FocalID,

rcov =~ idh(trait):units,

family = c("gaussian", "gaussian"),

prior = prior_follow,

nitt = 1000000,

burnin = 50000,

thin = 300,

verbose = TRUE,

pr = TRUE,

data = as.data.frame(influencer))

summary(mcmc.follower.boldness)

follower_boldness <- mcmc.follower.boldness$VCV[,"traitlog_Bold_FID:traitlog.Follower.FocalID"]/

(sqrt(mcmc.follower.boldness$VCV[,"traitlog_Bold_FID:traitlog_Bold_FID.FocalID"])*

sqrt(mcmc.follower.boldness$VCV[,"traitlog.Follower:traitlog.Follower.FocalID"]))

mean(follower_boldness)

HPDinterval(follower_boldness)

#credible intervals overlap zero

# Model diagnostics

plot.MCMCglmm(mcmc.follower.boldness)

plot(mcmc.follower.boldness$Sol)

plot(mcmc.follower.boldness$VCV)

autocorr(mcmc.follower.boldness$VCV)[,,"traitlog_Bold_FID:traitlog_Bold_FID.FocalID"][3,4]

autocorr(mcmc.follower.boldness$VCV)[,,"traitlog.Follower:traitlog.Follower.FocalID"][3,4]

# Alternative model run #1

mcmc.follower.boldness1 <- MCMCglmm(cbind(log.Follower, log_Bold_FID) ~ trait

+ trait:Focal_Sex,

random =~ us(trait):FocalID,

rcov =~ idh(trait):units,

family = c("gaussian", "gaussian"),

prior = prior_follow,

nitt = 1000000,

burnin = 50000,

thin = 300,

verbose = TRUE,

pr = TRUE,

data = as.data.frame(influencer))

# Alternative model run #2

mcmc.follower.boldness2 <- MCMCglmm(cbind(log.Follower, log_Bold_FID) ~ trait

+ trait:Focal_Sex,

random =~ us(trait):FocalID,

rcov =~ idh(trait):units,

family = c("gaussian", "gaussian"),

prior = prior_follow,

nitt = 1000000,

burnin = 50000,

thin = 300,

verbose = TRUE,

pr = TRUE,

data = as.data.frame(influencer))

chainList <- mcmc.list(mcmc.follower.boldness$Sol,mcmc.follower.boldness1$Sol, mcmc.follower.boldness2$Sol)

plot(chainList)

gelman.diag(chainList)

gelman.plot(chainList)

### Followers and Aggressiveness ###

mcmc.follower.aggr <- MCMCglmm(cbind(log.Follower, Aggr_Min_Dist) ~ trait

+ trait:Focal_Sex,

random =~ us(trait):FocalID,

rcov =~ idh(trait):units,

family = c("gaussian", "gaussian"),

prior = prior_follow,

nitt = 1000000,

burnin = 50000,

thin = 300,

verbose = TRUE,

pr = TRUE,

data = as.data.frame(influencer))

summary(mcmc.follower.aggr)

follower_aggr <- mcmc.follower.aggr$VCV[,"traitAggr_Min_Dist:traitlog.Follower.FocalID"]/

(sqrt(mcmc.follower.aggr$VCV[,"traitAggr_Min_Dist:traitAggr_Min_Dist.FocalID"])*

sqrt(mcmc.follower.aggr$VCV[,"traitlog.Follower:traitlog.Follower.FocalID"]))

mean(follower_aggr)

HPDinterval(follower_aggr)

#credible intervals overlap zero

# Model diagnostics

plot.MCMCglmm(mcmc.follower.aggr)

plot(mcmc.follower.aggr$Sol)

plot(mcmc.follower.aggr$VCV)

autocorr(mcmc.follower.aggr$VCV)[,,"traitAggr_Min_Dist:traitAggr_Min_Dist.FocalID"][3,4]

autocorr(mcmc.follower.aggr$VCV)[,,"traitlog.Follower:traitlog.Follower.FocalID"][3,4]

# Alternative model run #1

mcmc.follower.aggr1 <- MCMCglmm(cbind(log.Follower, Aggr_Min_Dist) ~ trait

+ trait:Focal_Sex,

random =~ us(trait):FocalID,

rcov =~ idh(trait):units,

family = c("gaussian", "gaussian"),

prior = prior_follow,

nitt = 1000000,

burnin = 50000,

thin = 300,

verbose = TRUE,

pr = TRUE,

data = as.data.frame(influencer))

# Alternative model run #2

mcmc.follower.aggr2 <- MCMCglmm(cbind(log.Follower, Aggr_Min_Dist) ~ trait

+ trait:Focal_Sex,

random =~ us(trait):FocalID,

rcov =~ idh(trait):units,

family = c("gaussian", "gaussian"),

prior = prior_follow,

nitt = 1000000,

burnin = 50000,

thin = 300,

verbose = TRUE,

pr = TRUE,

data = as.data.frame(influencer))

chainList <- mcmc.list(mcmc.follower.aggr$Sol,mcmc.follower.aggr1$Sol, mcmc.follower.aggr2$Sol)

plot(chainList)

gelman.diag(chainList)

gelman.plot(chainList)

### Followers and Neophilia ###

mcmc.follower.neo <- MCMCglmm(cbind(log.Follower, Neo_Delta_Latency) ~ trait

+ trait:Focal_Sex,

random =~ us(trait):FocalID,

rcov =~ idh(trait):units,

family = c("gaussian", "gaussian"),

prior = prior_follow,

nitt = 1000000,

burnin = 50000,

thin = 300,

verbose = TRUE,

pr = TRUE,

data = as.data.frame(influencer))

summary(mcmc.follower.neo)

follower_neo <- mcmc.follower.neo$VCV[,"traitNeo_Delta_Latency:traitlog.Follower.FocalID"]/

(sqrt(mcmc.follower.neo$VCV[,"traitNeo_Delta_Latency:traitNeo_Delta_Latency.FocalID"])*

sqrt(mcmc.follower.neo$VCV[,"traitlog.Follower:traitlog.Follower.FocalID"]))

mean(follower_neo)

HPDinterval(follower_neo)

# Model diagnostics

plot.MCMCglmm(mcmc.follower.neo)

plot(mcmc.follower.neo$Sol)

plot(mcmc.follower.neo$VCV)

autocorr(mcmc.follower.neo$VCV)[,,"traitNeo_Delta_Latency:traitNeo_Delta_Latency.FocalID"][3,4]

autocorr(mcmc.follower.neo$VCV)[,,"traitlog.Follower:traitlog.Follower.FocalID"][3,4]

# Alternative model run #1

mcmc.follower.neo1 <- MCMCglmm(cbind(log.Follower, Neo_Delta_Latency) ~ trait,

+ trait:Focal_Sex,

random =~ us(trait):FocalID,

rcov =~ idh(trait):units,

family = c("gaussian", "gaussian"),

prior = prior_follow,

nitt = 1000000,

burnin = 50000,

thin = 300,

verbose = TRUE,

pr = TRUE,

data = as.data.frame(influencer))

# Alternative model run #2

mcmc.follower.neo2 <- MCMCglmm(cbind(log.Follower, Neo_Delta_Latency) ~ trait,

+ trait:Focal_Sex,

random =~ us(trait):FocalID,

rcov =~ idh(trait):units,

family = c("gaussian", "gaussian"),

prior = prior_follow,

nitt = 1000000,

burnin = 50000,

thin = 300,

verbose = TRUE,

pr = TRUE,

data = as.data.frame(influencer))

chainList <- mcmc.list(mcmc.follower.neo$Sol,mcmc.follower.neo1$Sol, mcmc.follower.neo2$Sol)

plot(chainList)

gelman.diag(chainList)

gelman.plot(chainList)

##### Figure 3 #####

# Boldness

boldness_follower_cors <- tibble(Traits = c("log_Bold_FID, log.Follower"),

Estimate = mean(follower_boldness),

Lower = c(HPDinterval(follower_boldness)[,"lower"]),

Upper = c(HPDinterval(follower_boldness)[,"upper"]))

boldness_follower_plot <- ggplot(boldness_follower_cors, aes(x = Traits, y = Estimate)) +

geom_pointrange(aes(ymin = Lower, ymax = Upper)) +

geom_hline(yintercept = 0, linetype = "dotted", alpha = 0.3) +

scale_x_discrete(labels = element_blank()) +

theme_classic() +

theme(panel.border = element_rect(colour = "black", fill=NA), panel.background = element_rect(fill="white"), panel.grid.major = element_blank(),

panel.grid.minor = element_blank(), axis.line = element_line(colour = "black"),

axis.title.x = element_text(colour="black", size=14),

axis.title.y = element_text(colour="black", size=14, angle = 0, vjust = 0.5)) +

labs(x = " Boldness",

y = "") +

ylim(-1,1) + coord_flip()

boldness_follower_plot

# Aggressiveness

aggr_follower_cors<- tibble(Traits = c("Aggr_Min_Dist, log.Follower"),

Estimate = mean(follower_aggr),

Lower = c(HPDinterval(follower_aggr)[,"lower"]),

Upper = c(HPDinterval(follower_aggr)[,"upper"]))

aggr_follower_plot <- ggplot(aggr_follower_cors, aes(x = Traits, y = Estimate)) +

geom_pointrange(aes(ymin = Lower, ymax = Upper)) +

geom_hline(yintercept = 0, linetype = "dotted", alpha = 0.3) +

scale_x_discrete(labels = element_blank()) +

theme_classic() +

theme(panel.border = element_rect(colour = "black", fill=NA), panel.background = element_rect(fill="white"), panel.grid.major = element_blank(),

panel.grid.minor = element_blank(), axis.line = element_line(colour = "black"),

axis.title.x = element_text(colour="black", size=14),

axis.title.y = element_text(colour="black", size=14, angle = 0, vjust = 0.5)) +

labs(x = "Aggressiveness",

y = "") +

ylim(-1,1) + coord_flip()

aggr_follower_plot

# Neophobia

neo_follower_cors<- tibble(Traits = c("Neo_Delta_Latency, log.Follower"),

Estimate = mean(follower_neo),

Lower = c(HPDinterval(follower_neo)[,"lower"]),

Upper = c(HPDinterval(follower_neo)[,"upper"]))

neo_follower_plot <- ggplot(neo_follower_cors, aes(x = Traits, y = Estimate)) + coord_flip() +

geom_pointrange(aes(ymin = Lower, ymax = Upper)) +

geom_hline(yintercept = 0, linetype = "dotted", alpha = 0.3) +

scale_x_discrete(labels = element_blank()) +

theme_classic() +

theme(panel.border = element_rect(colour = "black", fill=NA), panel.background = element_rect(fill="white"), panel.grid.major = element_blank(),

panel.grid.minor = element_blank(), axis.line = element_line(colour = "black"),

axis.title.x = element_text(colour="black", size=14),

axis.title.y = element_text(colour="black", size=14, angle = 0, vjust = 0.5)) +

labs(x = " Neophobia",

y = "Correlation (Estimate +/- 95% CIs)") +

ylim(-1,1)

neo_follower_plot

# Arrange

Figure3 <- ggarrange(boldness_follower_plot, aggr_follower_plot, neo_follower_plot, nrow=3, ncol=1, labels = c("a)", "b)", "c)"))

Figure3

##### Figure S2 #####

ggplot(influencer, aes(x = Group_Size)) +

geom_histogram(breaks = seq(min(forHist$SubgroupSize) - 0.5, max(forHist$SubgroupSize) + 0.5, by = 1), fill = "skyblue", color = "black") +

labs(x = "Group Size", y = "Frequency", title = "") +

theme_classic() +

theme(axis.text = element_text(color = "black", size = 24), title = element_text(size = 30), strip.text = element_text(size = 24),

panel.grid = element_blank(), axis.line = element_line(color = "black"), panel.spacing.x = unit(0, "lines"),

text = element_text(color = "black"), axis.title = element_text(color = "black", size = 30)) +

xlim(0,40)
